# Comparative Transcriptomics and Proteomics Analyses of Leaves Reveals a Freezing Stress-Responsive Molecular Network in Winter Rapeseed (*Brassica rapa* L.)

**DOI:** 10.3389/fpls.2021.664311

**Published:** 2021-04-28

**Authors:** Jiaping Wei, Guoqiang Zheng, Xingwang Yu, Sushuang Liu, Xiaoyun Dong, Xiaodong Cao, Xinling Fang, Hui Li, Jiaojiao Jin, Wenbo Mi, Zigang Liu

**Affiliations:** ^1^Gansu Province Key Laboratory of Aridland Crop Sciences, Lanzhou, China; ^2^College of Agronomy, Gansu Agricultural University, Lanzhou, China; ^3^Department of Crop and Soil Sciences, North Carolina State University, Raleigh, NC, United States; ^4^Department of Life Sciences and Health, Huzhou University, Huzhou, China

**Keywords:** winter rapeseed, freezing stress, transcript, protein, freezing tolerance, physiology and biochemistry

## Abstract

Winter rapeseed is susceptible to low temperature during winter in Northwest China, which could lead to a severe reduction of crop production. The freezing temperature could stress the whole plant, especially the leaf, and ultimately harm the survival rate of winter rapeseed. However, the molecular mechanism underlying freezing tolerance is still unclear in winter rapeseed. In this study, a comprehensive investigation of winter rapeseed freezing tolerance was conducted at the levels of transcript, protein, and physiology and biochemistry, using a pair of freezing-sensitive and freezing-resistant cultivars NQF24 and 17NTS57. There were 4,319 unique differentially expressed genes (DEGs) and 137 unique differentially abundant proteins (DAPs) between two cultivars identified in leaf under freezing stress. Function enrichment analysis showed that most of the enriched DEGs and DAPs were involved in plant hormone signal transduction, alpha-linolenic/linoleic acid metabolism, peroxisome, glutathione metabolism, fatty acid degradation, and secondary metabolite biosynthesis pathways. Based on our findings, it was speculated that freezing tolerance formation is caused by increased signal transduction, enhanced biosynthesis of protein, secondary metabolites, and plant hormones, elevated energy supply, greater reactive oxygen species scavenging, and lower lipid peroxidation as well as stronger cell stability in leaf under freezing stress. These results provide a comprehensive profile of leaf response under freezing stress, which have potential to be used as selection indicators of breeding programs to improve freezing tolerance in rapeseed.

## Introduction

Cold stress is categorized into chilling stress (0–15°C) and freezing stress (<0°C) in crops, which imposes deleterious effects on plant survival, growth, and development, restricts the geographical distribution of plant species, and influences crop production (Chinnusamy et al., 2007; Ding et al., 2019). Freezing stress is one of the most important environmental stresses in Northwest China from late autumn to early spring, which could not only reduce the yield but also harm the quality of economically important crops, especially for winter crops grown in high-latitude and high-altitude regions (Xu et al., 2018). Moreover, freezing stress damages the stability of proteins or protein complexes and reduces the activities of reactive oxygen species (ROS)-scavenging enzymes during seed development (Zeng et al., 2018).

Winter rapeseed (*Brassica napus* L.) is a fundamental and nutritious oilseed crop that is widely planted in Northwest China. However, the growth and the development of winter rapeseed are vulnerable to several factors, such as extremely low temperature, low level of precipitation, and heavy evaporation in the region, which affect the normal overwintering and propagation of rapeseed, leading to a reduction of yield and quality. Hence, there is a high demand to develop rapeseed cultivars with strong freezing tolerance to withstand the stress for overwinter safely.

So far, abundant studies have been focused on cold tolerance response in model plants, such as *Arabidopsis* and rice (Ding et al., 2015; Zhang et al., 2017). However, the molecular mechanism underlying freezing response in winter rapeseed is far away lagging behind. Recently, Zeng et al. (2018) suggested the impacts on the roots of two rapeseed cultivars with different freezing tolerance under freezing stress by isobaric tags for relative and absolute quantification (iTRAQ). Xu et al. (2018) and Pu et al. (2019) expounded the effects on the freezing tolerance formation in the leaves of two rapeseed cultivars with different freezing tolerance under cold stress by iTRAQ and transcriptome sequencing, respectively. Nevertheless, most studies only investigated single omics response based on traditional data-dependent acquisition (DDA) models. In the recent years, data-independent acquisition (DIA) technology is becoming popular due to its extremely high detection rate, enabling the identification and quantification of almost all protein molecules in complex samples. In addition, the quantitative analysis of protein DIA is also rated as one of the most noteworthy technologies by the journal *Nature Methods* (Egertson et al., 2013).

In the present study, we comprehensively analyzed the physiological and structural characteristics of a pair of resistant and susceptible winter rapeseed cultivars under freezing stress, which include determination of the antioxidant enzyme activities, net photosynthetic rate, and microscopic observation. Meanwhile, the combination of RNA sequencing-based transcriptomics and DIA-based proteomics was employed to identify differentially expressed genes’ (DEGs) and differentially abundant proteins’ (DAPs) response to freezing tolerance between two winter rapeseed cultivars. Subsequently, the main metabolic pathways and cellular processes that participated in freezing stress were explored. The findings will expand the understanding of the molecular regulatory network by which winter rapeseed responds to cold stress.

## Materials and Methods

### Plant Samples and Freezing Stress Treatments

The two extension winter rapeseed cultivars in Northwest China, freezing-resistant cultivar 17NTS57 (NS, with more than 85% overwinter survival rate at −26°C, dark brown seed, 33.02% oleic acid, and 17.29% linoleic acid) and freezing-sensitive cultivar NQF24 (NF, with 0% overwinter survival rate below −10°C, dark brown seed, 36.98% oleic acid, and 16.73% linoleic acid), used in this study were provided by Gansu Agricultural University (Mi et al., 2021). The seedlings of the two cultivars were grown in plastic pots (5 L) filled with soil and normally maintained. At four-leaf stage, the potted plants were divided into two groups. The treatment group (treatment, T1) was transferred to a chamber [−4°C, 60% humidity, 12/12 h (day/night) photoperiod, and 350 μmol m^−2^·s^−1^ irradiance] for 12 h, while the control group (control, T0) was maintained in normal condition [22°C, 60% humidity, 12/12 h (day/night) photoperiod, and 350 μmol m^−2^·s^−1^ irradiance]. The second leaf was sampled from the control and the treated plants, frozen in liquid nitrogen immediately, and stored at −80°C for RNA and protein extraction and determination of physiological and biochemical characteristics. The experiment was conducted in three independent biological replicates.

### Evaluation of Physiological and Biochemical Characteristics

The measurement of net photosynthetic rate was carried out as per Chu et al. (2015), with minor modifications. After freezing stress, the third fresh leaves of both cultivars were used for photosynthesis evaluation, utilizing a LI-COR 6400 portable gas analysis system (Lincoln, NE, United States) setting a built-in light source of 1,000 μmol photons m^−2^·s^−1^. Each selected sample was measured at least four times at 25°C. All determinations were operated at 9:00 am to 11:00 am to minimize the error.

A total of 0.2 g rapeseed leaves was used to measure the physiological and biochemical characteristics, including ROS-scavenging enzyme activities, lipid peroxidation level, free proline content, and relative electrolytic leakage. The ROS-scavenging enzyme activities were measured using kits from Beijing Solarbio Science and Technology, following the manufacturer’s protocols (Chen et al., 2019). The samples were homogenized using 1 ml 0.05 mol/L phosphate-buffered saline extraction buffer (pH 7.8). The supernatant was centrifuged at 4°C for 15 min at 8,000 × *g* and used for catalase (CAT), superoxide dismutase (SOD), and peroxidase (POD) activity analysis using Thermo Multiscan FC. The level of lipid peroxidation was evaluated by determining the malondialdehyde (MDA) content according to Wei et al. (2020). The samples were fully ground and extracted in a buffer of 0.67% thiobarbituric acid in 20% trichloroacetic acid (TCA) at 4°C and then centrifuged at 12,000 × *g* for 20 min. The absorbance of each sample supernatant was measured at 450, 532, and 600 nm.

The proline measurement was executed as described by Zhang et al. (2018); 0.2 g rapeseed leaves from treatments and controls was ground in liquid nitrogen and extracted by the proline assay kit (Solarbio). After centrifugation at 12,000 × *g* for 20 min, the supernatants were used for proline analysis with a spectrometer. The relative electrolyte leakage (REL) was measured as described by Huang et al. (2017b); 0.1 g rapeseed leaves from treatments and controls was cut into 2 cm × 2-cm segments and were transferred into a 50-ml centrifuge tube that was filled with 15 ml of deionized water. The centrifuge tube with fragments was shaken for 24 h at room temperature, and the primary conductivity (EL1) was measured with a conductivity meter. Subsequently, the leaves in the tube were heated at 121°C for 20 min to disrupt the tissues and release all the electrolytes into the solution completely. After the samples had cooled to room temperature, the second conductivity (EL2) was measured. The relative EL was calculated by using the equation: EL = (EL1/EL2) × 100%. All measurements were performed with at least three biological replications.

### Transmission Electron Microscope Observations

For transmission electron microscope (TEM) observations, fresh rapeseed leaves were cut into 2 mm × 2-mm sections and fixed in 2.5% glutaraldehyde solution for 16 h at 4°C, followed by fixation with 1% KMnO_4_ solution for 2 h. All the fixed samples were dehydrated in a graded series of ethanol and embedded in Spurr resin. Eighty-nanometer-thick ultrathin sections were finally stained with uranyl acetate for 15 min and lead citrate for 10 min. Observations were operated on an H-7650 electron microscope. The construction of high-quality TEM images was manipulated as described by Toyooka et al. (2014).

### Rapeseed Leaf RNA Extraction, RNA Sequencing, and Quantitative Real-Time PCR

Total RNAs from four samples containing three biological replicates were ground in liquid nitrogen and extracted using TRIzol Reagent (Tiangen Biotech, China) according to the manufacturer’s instructions. The library construction and sequencing were performed by Gene Denovo Biotechnology Co. (Guangzhou, China) on an Illumina HiSeqTM 2500 platform. After the Illumina sequencing, three replicates of raw sequences for each sample were filtered to generate clean reads for subsequent analysis. The expressions of 12 candidate freezing-responsive genes and proteins were analyzed by qRT-PCR. The qRT-PCR was performed according to the protocol of Wei et al. (2020). All the primers are listed in Supplementary Table S1. The relative quantification (2^−ΔΔCt^) of gene expression was evaluated using comparative cycle threshold method, and each sample was replicated three times.

### Filtering of Reads and Gene Expression Analysis

Clean data were obtained by removing the reads containing adapters, reads containing poly-N, and low-quality reads from raw data. The high-quality paired-end reads from each sample were mapped to rapeseed reference genome by TopHat v2.0.3.12 as illustrated (Kim et al., 2013b). The gene expression levels were calculated and normalized as fragments per kilobase per million mapped reads, which can be directly used for identifying DEGs in pair-wise comparisons (Trapnell et al., 2010). Genes with a *p*-value <0.001 and a value of |log_2_foldchange| ≥2 by the edgeR package were assigned as DEGs.^1^ The sequenced transcriptome raw data have been deposited to the Sequence Read Archive at NCBI with accession number PRJNA685002.

### Protein Extraction and Digestion

Protein extraction was conducted using the method of cold TCA/acetone precipitation according to Wei et al. (2020), with minor modifications. Plant leaves (0.2 g) from four samples containing three biological replicates were ground to powder in liquid nitrogen and then dissolved in 2 ml lysis buffer containing 7 M urea, 2% SDS, 1 × protease inhibitor cocktail (Roche Ltd. Basel, Switzerland), followed by sonication on ice for 20 min and centrifugation at 12,000 rpm for 10 min at 4°C. The supernatant was transferred into a fresh tube. For each sample, the proteins were precipitated with ice-cold acetone at −20°C overnight. The precipitations were cleaned with acetone three times and re-dissolved in 8 M urea and homogenized for 3 min in ice using an ultrasonic homogenizer. The homogenate was centrifuged at 12,000 rpm for 15 min at 4°C. The supernatant was collected, and the protein concentration was measured by the BCA Protein Assay Kit (Thermo Fisher Scientific, Waltham, MA). Ten, 50 μg proteins extracted from leaves was suspended in 50 μl of 50 mM ammonium bicarbonate, reduced by adding 10 ul of 100 mM dithiothreitol for 1 h at 55°C and alkylated by adding 5 ul of 20 mM iodoacetamide for 1 h at 37°C in the dark. Subsequently, protein samples were precipitated using 300 ul pre-chilled acetone at −20°C overnight. The precipitates were washed twice with cold acetone and resuspended in 50 mM ammonium bicarbonate. The samples were digested with sequencing-grade modified trypsin (Promega, Madison, WI) at an enzyme/substrate ratio of 1:50 (w/w) for 16 h at 37°C.

### High-pH Reverse-Phase Separation and Liquid Chromatography With Tandem Mass Spectrometry Analysis

The digested peptides were separated on an Ultimate 3,000 system (Thermo Fisher Scientific, United States) with a high-pH reverse-phase XBridge BEH C18 column (4.6 mm × 250 mm, 5 μm) from Waters. The peptides were eluted at a flow rate of 1 ml/min. Buffer A consisted of 20 mM ammonium formate in water (pH 10.0), and buffer B consisted of 20 mM ammonium formate in 80% v/v acetonitrile (pH 10.0). High pH separation was performed using a linear gradient, starting from 5% B to 45% B in 40 min. For each sample, 10 fractions were collected and dried in vacuum centrifugation. All the samples were stored at −80°C until further analysis.

The peptides were dissolved in 30 μl buffer which consisted of 0.1% formic acid in water and 0.1% formic acid in acetonitrile (buffer D). Liquid chromatography with tandem mass spectrometry analysis was carried out on an Orbitrap Fusion Lumos mass spectrometer (Thermo Fisher Scientific, Germany) coupled with a Nano ACQUITY UPLC system (Waters Corporation, Milford, MA). Briefly, 10 μl of peptides was loaded onto a trapping column (Acclaim PepMap C18, 100 μm × 2 cm) and then eluted on an analytical column (Acclaim PepMap C18, 75 μm × 25 cm) by a 120-min gradient of buffer D. The column flow rate was maintained at 500 nl/min, and electrospray voltage of 2.1 kV was used.

For library generation, the Orbitrap Fusion Lumos was operated in positive mode to acquire data in DDA mode. Mass spectrometry (MS) spectra (350–1,500 m/z) were collected at 1.2 × 10^5^ resolution to reach an automatic gain control (AGC) target of 4.0 × 10^5^. The maximum injection time was set to 50 ms. The precursor ions were used for MS/MS detection using a normalized collision energy of 32. Dynamic exclusion was set to 30 s to exclude all isotopes. MS/MS spectra were collected at 1.5 × 10^4^ resolution to reach an AGC target of 5.0 × 10^4^ with a maximum injection time of 35 ms. For data acquisition, DIA was used. MS spectra (350–1,500 m/z) were collected at 1.2 × 10^5^ resolution to reach an AGC target of 5.0 × 10^5^. The maximum injection time was set to 50 ms. MS/MS spectra were collected at 3.0 × 10^4^ resolution to reach an AGC target of 1.0 × 10^6^ with a collision energy of 32. A total of 60 segments were selected for MS/MS using normalized collision energy of 32. The acquisition window covered 1 m/z through 60 consecutive isolation windows.

### Mass Spectra Data and Protein Quantification

Spectronaut Pulsar 11.0 (Biognosys AG) software (Bruderer et al., 2015) was used to analyze and process the DDA MS/MS data with the following settings: trypsin as the digestion enzyme, carbamidomethylation (C) as the fixed modification, oxidation (M) as the variable modifications, 20 ppm as precursor mass tolerance, and 0.05 Da as fragment mass tolerance. The MS/MS data were searched from the database of *B. napus*, which was downloaded from the NCBI (*Brassica napus* v2.0, 123,465 entries; Supplementary Table S1). False discovery rate of peptides was dynamically set at 1%, which was calculated by a reverse search sequence. Subsequently, the raw data of every sample with the spectra library based on DIA MS/MS data were analyzed by Spectronaut Pulsar 11.0 with the default parameters depending on the previously constructed DDA protein dataset, which included protein sequences (19,394), precursors (49,118), and peptides (39,809). All protein quantification normalizations were performed by local normalization with the Pulsar software. The mass spectrometry data have been submitted into a public iProX database (Ma et al., 2019) with accession number IPX0002682000. Proteins with a value of *p* < 0.001, and peptides with a value of *p* ≥ 2 and a value of |log_2_foldchange| ≥1 by the edgeR package were filtered as DAPs (see Footnote 1).

### Bioinformatics and Statistical Analysis

The correlation coefficient between three replicates was calculated to evaluate the repeatability of the experimental results between samples. Principal component analysis (PCA) was performed to reveal the structure/relationship of samples by R package gmodels (see Footnote 1). The proteins and genes were annotated against Gene Ontology (GO) database and Kyoto Encyclopedia of Genes and Genomes (KEGG) databases (Binns et al., 2009; Kanehisa et al., 2016).^2^^,^^3^ Enrichment analysis was performed based on GO and KEGG databases. Physiological data were subjected to one-way analyses of variance, and the mean differences of three replicates were compared by least significant difference test.

## Results

### Physiological and Biochemical Responses to Freezing Stress

**Figure 1:**
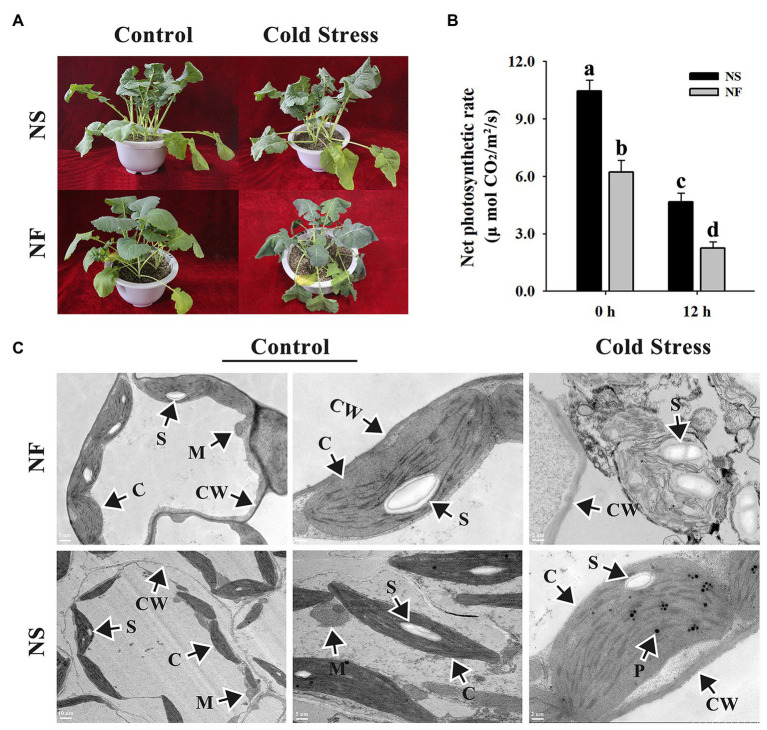
Morphological and photosynthetic changes in the leaves of winter rapeseed under freezing stress. **(A)** Illustration of 5-week-old winter rapeseed plants under freezing treatment. **(B)** The net photosynthetic rates of rapeseed cultivars NF and NS under freezing treatment. Values of *p* are means ± SD from three biological replicates (*p* < 0.05). **(C)** Microstructure changes in the leaves of both cultivars under freezing treatment for 12 h and control, respectively. C, chloroplast; CW, cell wall; M, mitochondrion; P, plastoglobulus; S, starch grain.

After the freezing treatment, NF plants appeared more severely wilting than NS plants (Figure 1A). Freezing stress caused a significant decrease in the net photosynthetic rates of both rapeseed cultivars in comparison with the normal condition, whereas under freezing stress, the net photosynthetic rate of rapeseed cultivar NS was significantly (*p* < 0.05) higher than that of cultivar NF. Interestingly, if we do the relative values on net photosynthesis, we discovered that the net photosynthetic rate decreased for approximately 50% in both cases (Figure 1B). Through TEM analysis, the chloroplasts in mesophyll cells were found to be disintegrated harshly in rapeseed cultivar NF after freezing stress, whereas more plastoglobuli were found in cells of rapeseed cultivar NS under freezing stress (Figure 1C). These results indicated that the freezing stress exerted greater damage to the leaf of rapeseed cultivar NF than to that of cultivar NS.

**Figure 2:**
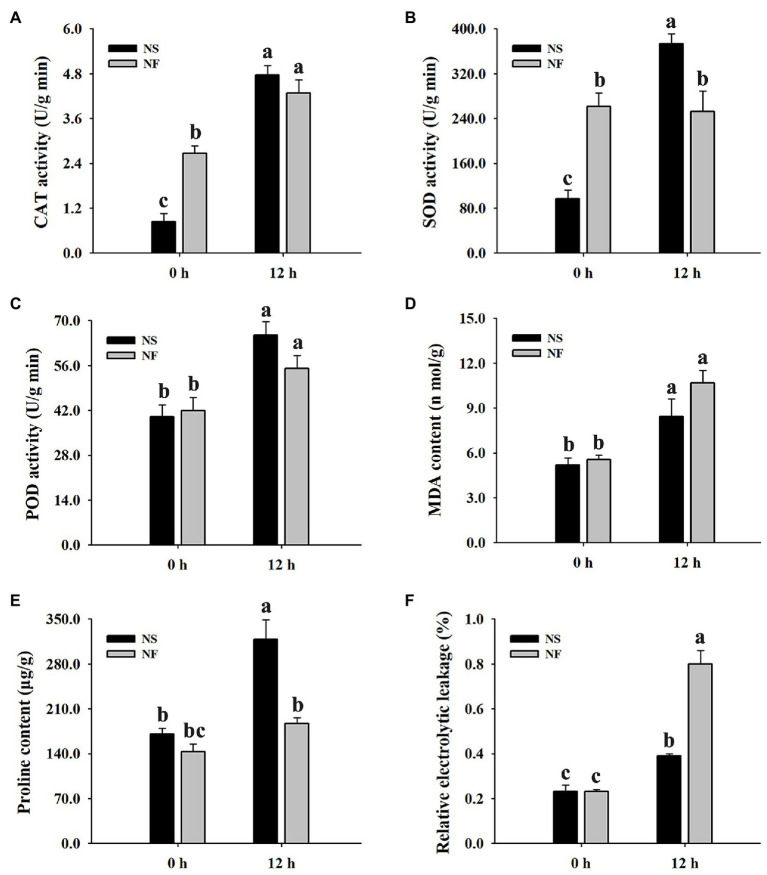
Enzyme activities of catalase (CAT), superoxide dismutase (SOD), and peroxidase (POD), malondialdehyde (MDA) content, free proline content, and relative electrolyte leakage (REL) in the leaves of winter rapeseed under freezing treatment for 0 and 12 h, respectively. **(A)** CAT activity, **(B)** SOD activity, **(C)** POD activity, **(D)** MDA content, **(E)** free proline content, **(F)** REL. Values of *p* are means ± SD from three biological replicates (*p* < 0.05).

The freezing stress caused a significant increase in the enzyme activities of CAT and POD in the leaf of both rapeseed cultivars. However, the enzyme activities of CAT, SOD, and POD in the leaf of cultivar NS maintained a higher level than those of cultivar NF under freezing stress (Figures 2A–C). Furthermore, the level of plasma membrane lipid peroxidation in the leaf of both cultivars was evaluated by measuring MDA and REL, respectively. Both MDA content and REL level were significantly (*p* < 0.05) increased in the leaf of both cultivars under freezing stress, but the levels in cultivar NF were significantly (*p* < 0.05) higher than those in cultivar NS (Figures 2D–F), indicating that the freezing treatment resulted in more severe plasma membrane damage in the leaf of cultivar NF than in that of cultivar NS. Moreover, free proline was significantly (*p* < 0.05) increased only in cultivar NS under freezing stress (Figure 2E). These results suggested that a high level of ROS scavenging capacity and a low level of lipid peroxidation might contribute to enhance freezing tolerance in winter rapeseed cultivar NS under freezing stress.

### Quality Control Analysis of Rapeseed Transcriptome and Proteome

**Figure 3:**
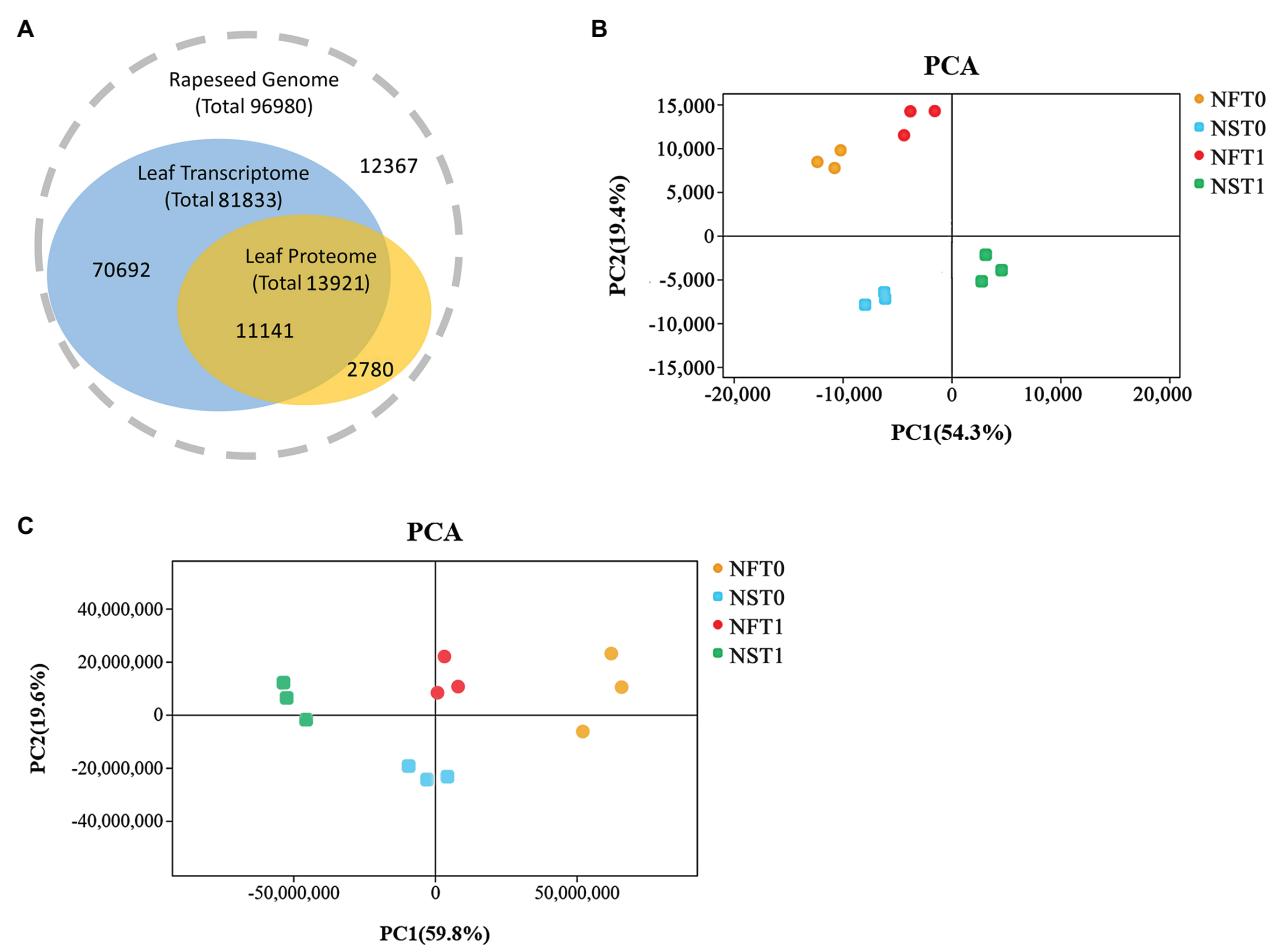
The overview of transcriptome and proteome in rapeseed cultivars NF and NS. **(A)** Consistency between the identified transcripts and proteins in the leaves of both cultivars. **(B)** Principal component analysis (PCA) of detected genes in RNA sequencing. **(C)** PCA of detected proteins in data-independent acquisition quantification.

Overall, transcripts were detected for 80.0% of the proteins (Figure 3A). The transcriptome analysis detected a total of 81,833 genes, including 75,034 known genes and 6,799 new genes in both rapeseed cultivars (Figure 3A; Supplementary Table S1). For the proteome analysis, a total of 30,471 unique peptides were identified from 38,159 spectra, which corresponded to 13,921 proteins and were encoded by 11,141 genes in transcriptome (Figure 3A; Supplementary Tables S1,S2). The PCA indicated that three biological replicates of NFT0, NFT1, NST0, and NST1 had good conformity (Figures 3B,C), and all biological replicates showed a high overlap in transcriptome and proteome, respectively (Supplementary Figure S1). In addition, a repeatability analysis between three biological replicates of NFT0, NFT1, NST0, and NST1 samples showed that their correlation coefficient was greater than 0.9 (Supplementary Figures S2, S3). These results indicated a high level of reliability of the transcriptome and proteome analyses.

### Quantitative Transcriptomic and Proteomic Analysis of Rapeseed Leaves Under Freezing Stress

**Figure 4:**
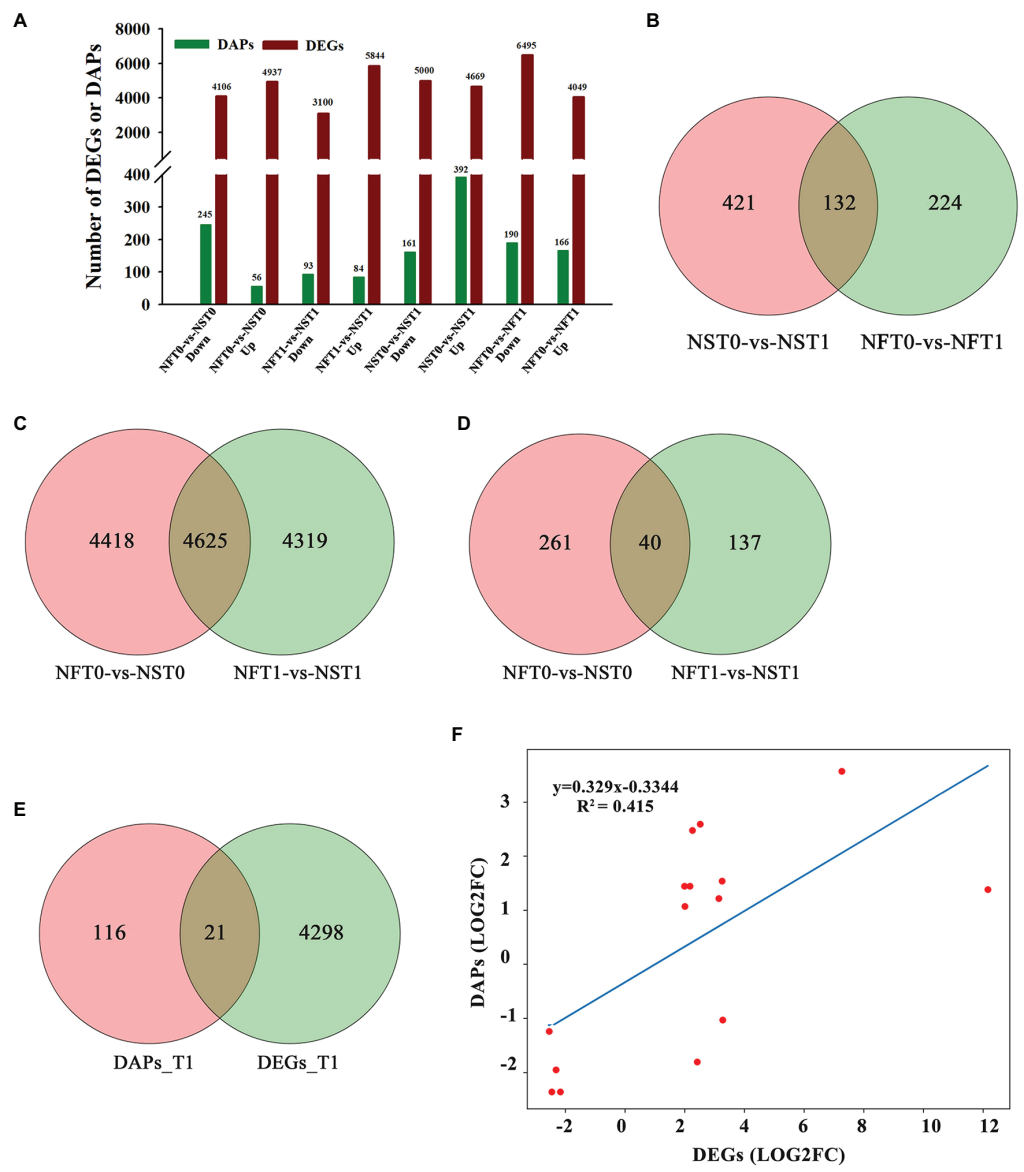
Comparison of transcript and protein abundance in developing rapeseed leaves. **(A)** The number of differentially expressed genes (DEGs) and differentially abundant proteins (DAPs) identified for two treatments in both cultivars. **(B)** DAPs between the control and freezing-stressed leaves in both cultivars. DEGs **(C)** and DAPs **(D)** identified in the leaf between both cultivars under freezing stress, respectively. **(E)** Venn diagram of the unique DEGs and DAPs between both cultivars at freezing treatment time of T1. **(F)** Concordance changes between DEGs and the encoded DAPs between both cultivars at freezing treatment time of T1.

To identify DEGs and DAPs that were induced by freezing treatment, the changes of transcription level and protein relative abundance in leaves of both cultivars were analyzed through comparison of T1 to T0. Compared to the corresponding controls, a total of 9,669 (up, 4,669; down, 5,000) genes and 10,544 (up, 4,049; down, 6,495) genes were identified to be differentially expressed in the leaves of rapeseed cultivars NS and NF under freezing stress, respectively (Figure 4A; Supplementary Table S3). In comparison with controls, a total of 392 and 166 proteins were found to be accumulated in abundance in the leaves of cultivars NS and NF under freezing stress, respectively, whereas 161 and 190 proteins were reduced in abundance in cultivars NS and NF under freezing stress, respectively (Figure 4A; Supplementary Table S4). It is worth noting that the number of DEGs and DAPs was significantly changed in both cultivars, implying a greatly diverse dynamics of gene or protein expression regulation in both cultivars. In addition, more accumulated proteins were found in rapeseed cultivar NS than in NF, and a small number of DAPs were shared between both cultivars (Figure 4B).

### Comparative Analysis Between Protein Abundance and Gene Expression Levels

The relationship between the levels of transcription and protein would be evaluated. DAPs and DEGs were compared between both rapeseed cultivars at different freezing treatment time points (T0 and T1), respectively. A total of 4,625 shared DEGs and 40 shared DAPs were jointly owned in NFT0_NST0 and NFT1_NST1. Among the shared DEGs, 2,608 were up-regulated and 2,017 were down-regulated in NFT0_NST0, while 2,706 were up-regulated and 1,919 were down-regulated in NFT1_NST1 (Figure 4C; Supplementary Table S5). Among the shared DAPs, 25 were accumulated in abundance and 15 were reduced in both NFT0_NST0 and NFT1_NST1, and the accumulation trend of all proteins is exactly consistent (Figure 4D; Supplementary Table S5). Furthermore, 4,319 DEGs and 137 DAPs were identified only in NFT1_NST1 but not in NFT0_NST0 (Figures 4C,D; Supplementary Table S6), which were the molecular basis for the difference in freezing tolerance between both cultivars, and they were regarded as unique genes/proteins. In addition, 21 DAPs corresponding to DEGs were found in the common portion in transcriptome and proteome changes between both cultivars during freezing stress (Figure 4E; Supplementary Table S7), and the concordance changes between the gene and its encoded protein were analyzed using Pearson’s correlation test. The results indicated a significant positive correlation between the fold changes of DEGs and the corresponding DAPs, with a Pearson of 0.415 in NFT1_NST1 (Figure 4F). These results suggested that DEGs and their encoded DAPs play a collaborative role in the freezing tolerance of winter rapeseed.

### GO Annotation and KEGG Analysis of DEGs and DAPs Under Freezing Stress

**Figure 5:**
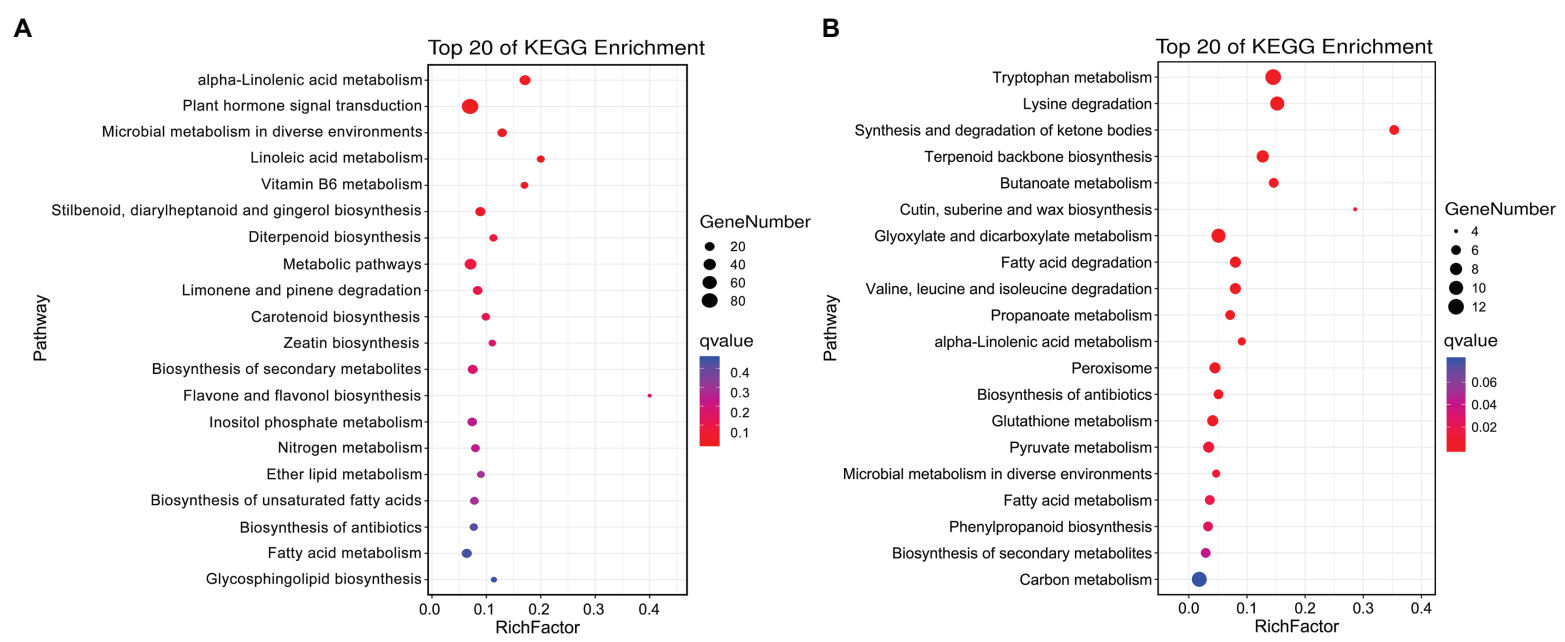
Kyoto Encyclopedia of Genes and Genomes (KEGG) pathway enrichment. Top 20 KEGG pathways enriched by unique differentially expressed genes **(A)** and differentially abundant proteins **(B)** between both rapeseed cultivars at freezing treatment time of T1.

The specific DEGs and DAPs in NST1_NFT1 were subjected to GO classification annotation (Supplementary Figure S4; Supplementary Table S6) and KEGG analysis. In total, 126 metabolic pathways were altered. The top 20 KEGG enrichment pathways exhibited that alpha-linolenic acid metabolism (ko00592), plant hormone signal transduction (ko04075), microbial metabolism in diverse environments (ko01120), and linoleic acid metabolism (ko00591) pathways were significantly (*q* < 0.05) enriched by DEGs, while tryptophan metabolism (ko00380), alpha-linolenic acid metabolism (ko00592), peroxisome (ko04146), phenylpropanoid biosynthesis (ko00940), glutathione metabolism (ko00480), fatty acid degradation (ko00071), biosynthesis of secondary metabolites (ko01110), etc., were significantly (*q* < 0.05) enriched by DAPs (Figures 5A,B; Supplementary Table S8). These metabolic pathways were considered as candidate pathways that participated in the freezing tolerance of winter rapeseed.

### Differentially Expressed Transcription Factors Under Freezing Stress

**Figure 6:**
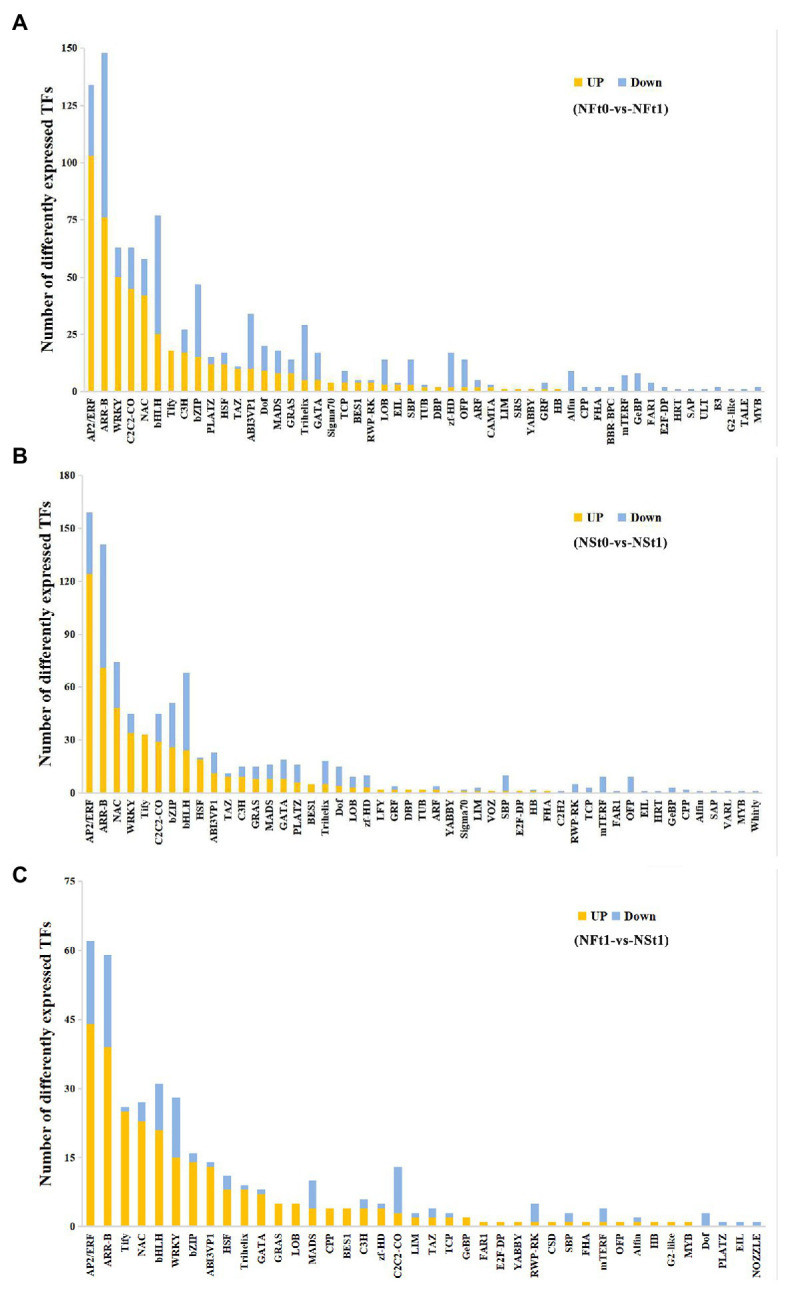
Classification of differentially expressed transcription factors (TFs) in developing rapeseed leaves. TFs identified in the leaf transcriptome of rapeseed cultivar NF **(A)** and NS **(B)** under freezing stress, respectively. **(C)** The unique TFs identified in leaf transcriptome between both cultivars at freezing treatment time of T1.

A total of 883 and 962 differentially expressed transcription factors (TFs) were identified as corresponding to the leaf transcriptome of cultivars NS and NF under freezing stress, respectively (Supplementary Table S9). In both cultivars, most of the up-regulated TFs belonged to ethylene response factor (AP2/ERF), NAC domain-containing protein (NAC), WRKY transcription factor (WRKY), heat stress transcription factor (HSF), TAZ domain-containing protein (TAZ), protein TIFY (TIFY), and zinc finger protein (C2C2) families, while most of the down-regulated TFs belonged to the transcription factor bHLH (bHLH), zinc-finger homeodomain protein (zf-HD), squamosa promoter-binding-like protein (SBP), transcription repressor OFP (OFP), and trihelix transcription factor (Trihelix) families (Figures 6A,B). Contrary to the findings of transcriptome, we did not find many differentially expressed TFs in proteome. There were only two and six TFs identified in the leaf proteome of cultivars NS and NF under freezing stress, respectively, and seven out of eight TFs were reduced in abundance. These results indicated the complicated dynamic changes between gene expression regulation and protein expression regulation.

In addition, a TF analysis of unique DEGs in NST1_NFT1 was executed. Generally, in line with the above-mentioned studies, it was shown that most TFs classified into the AP2/ERF, NAC, WRKY, HSF, and TIFY families were up-regulated in cultivar NS under freezing stress compared to cultivar NF (Figure 6C; Supplementary Table S9). Our results suggested that these TFs in tolerant winter rapeseed cultivar NS are more susceptible to activation under freezing stress, thereby regulating the expression of downstream genes in response to freezing stress.

### RNA-Seq and DIA Quantitation Validation by qRT-PCR

**Figure 7:**
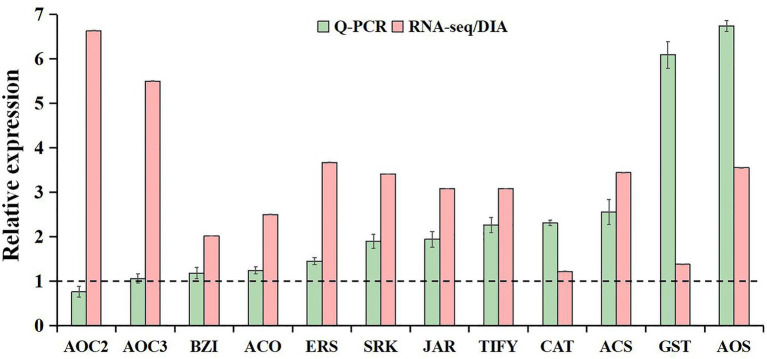
Comparative analysis of mRNA and RNA-seq or protein levels in leaves between rapeseed cultivar NF and cultivar NS under freezing stress. The data in the leaves of NF under freezing treatment were set to 1.0. Values are means ± SD (from three biological replicates) in NS leaves under freezing treatment.

The qRT-PCR analysis was used to validate the reliability of our transcriptome and proteome data. Based on high fold change, 12 freezing-responsive DEGs or DAPs were selected as targets. Among them, 11 out of 12 were found to be consistent at both the mRNA and RNA-Seq or protein levels under freezing stress in rapeseed cultivar NS compared to cultivar NF. Besides that, one gene showed an inconsistency between the mRNA and RNA-Seq levels (Figure 7). These results indicated that the qRT-PCR expression patterns were in good agreement with the RNA-Seq and DIA quantitation, and the RNA-Seq and DIA quantitation results were reliable in the present study.

## Discussion

It is generally believed that chilling and freezing stresses lead to a considerable decline in productivity of winter rapeseed, which is common in many winter rapeseed production areas around China (Zhu, 2016). Leaves, as the primary organ for photosynthetic accumulation products during cold acclimation, are crucial for overwinter survival rate and the reconstruction of above-ground organ after the revival stage once it perceives cold signaling, followed rapidly with response to cold stress, which would increase the cold tolerance and maintain its function. Some previous studies have also investigated the effects of freezing stress on winter rapeseed leaf tolerance formation by proteomics (Xu et al., 2018; Zeng et al., 2018), transcriptome, and physiology (Pu et al., 2019). However, the current unraveling of the intricate mechanism of the winter rapeseed in response to freezing stress remains elusive. The NS winter rapeseed can survive normally in winter in most northwestern areas from 38° to 42° north latitude, and its tolerance at low temperature environments is better than those of other types of winter rapeseed, which contain abundant cold-resistant genes. Therefore, in the present study, the thorough effects of cold stress on cold tolerance formation were investigated through the analysis of cold-tolerant NS and sensitive NF rapeseed leaf at the levels of transcript, protein, and physiology and biochemistry. It will be of great importance to elucidate the exhaustive mechanism of winter rapeseed for plant molecular breeding under cold stress.

### Physiological, Ultrastructural, and Photosynthetic Changes Between Both Winter Rapeseed Cultivars Under Freezing Stress

Cold stress can trigger a series of comprehensive physiological and biochemical events, leading to the synthesis of many substances or protective proteins in plants (Kaplan et al., 2007). Freezing stress provokes intracellular ice formation, which results in membrane lipid peroxidation and cell structural damage (Shi et al., 2018). For example, Gao et al. (2019) suggested that the level of lipid peroxidation in jojoba was significantly increased after cold treatment. Similarly, low electrolyte leakage and bad cell membrane integrity were exhibited in cold-stressed *Arabidopsis* roots (Deng et al., 2015). In this study, freezing stress gives rise to an increase in MDA content and REL level in the leaves of both cultivars, but the level of lipid peroxidation in cultivar NF was significantly higher than that in cultivar NS (Figures 2D,F). Coincidentally, the TEM micrographs exhibited a stronger cell ultrastructure stability in cultivar NS plants, compared with NF, after the freezing treatment (Figure 1C). Additionally, consistent with proteomic results, some beta tubulins said to contribute to cytoskeleton modifications were found to be accumulated in abundance (Supplementary Table S6), which implied that freezing stress induced rearrangements in the tubulin cytoskeleton, leading to the formation of a more stable cell structure (Livanos et al., 2014). In the past two decades, it has become increasingly evident that ROS plays a pivotal signal-regulating role in response to diverse abiotic stresses (Baxter et al., 2014). Under adverse environmental stresses, the ROS-scavenging enzymes are necessary to maintain normal cellular redox homeostasis (Ray et al., 2012). In the present study, ROS-scavenging enzyme activities were significantly increased in both cultivars under freezing stress, but the levels of enzyme activities in cultivar NS were higher than those in cultivar NF, and the enzyme activities of SOD were significantly different between NS and NF (Figure 2). Overall, these findings are in accordance with the findings reported by Zeng et al. (2018). Taken together, the enhanced ROS scavenging and the lower level of lipid peroxidation accompanied with more stable cell morphology in the leaves of winter rapeseed under freezing treatment would maintain a normal redox environment for leaf growth and development, thus leading to the stronger freezing tolerance in NS plants.

### Comparison of Effects of Freezing Stress on Freezing Tolerance Formation Between Both Winter Rapeseed Cultivars

A multitude of transcription factors have been reported in numerous higher plants, which monitor the expression of C-repeat binding transcription factor (CBF) by combining the corresponding cis-elements in their promoters under cold stress. After exposure to freezing stress, the CBF proteins rapidly recognize the promoter regions of downstream cold-regulated genes to activate their expression that enhances freezing tolerance (Jia et al., 2016; Zhao et al., 2016). For instance, bHLH transcription factor ICE1, calmodulin-binding transcription activator 3, brassinazole-resistant1 (BZR1), heat shock transcription factor C1 (HSFC1), and MYB88 /MYB124 transcription factors positively regulate the CBF expression at the transcriptional level, which contributes to cold stress (Kim et al., 2013a; Park et al., 2015; Li et al., 2017; Xie et al., 2018). In contrast, MYB15, phytochrome interacting factor3 (PIF3), PIF4, PIF7, and ethylene sensitivity3 negatively modulate the transcriptional repression of CBF (Shi et al., 2012; Jiang et al., 2017; Kim et al., 2017). In the present study, most of the AP2/ERF, NAC, WRKY, HSF, TAZ, TIFY, and C2C2 transcription factors were up-regulated in both rapeseed cultivars, while the transcription factors bHLH, zf-HD, SBP, OFP, and Trihelix were down-regulated (Figures 6A,B). Interestingly, these differentially expressed TFs encoded by genes were not found at the protein level, implying that those TFs might regulate the gene expression just at the transcription level, contributing to the freezing tolerance of winter rapeseed under freezing stress. Furthermore, over the past few years, several protein kinases including mitogen-activated protein kinases (MAPKs), Ca^2+^-dependent protein kinases (CDPKs), calcium/calmodulin-regulated receptor-like kinases (CRLKs), calcineurin-B-like interacting protein kinases (CIPKs), and receptor-like kinases (RLKs) have also been characterized to be crucial regulators of cold stress responses in plant (Liu et al., 2017; Zhao et al., 2017; Ding et al., 2019). In addition, a putative cold sensor chilling-tolerance divergence1 was reported to mediate a cold-sensing calcium channel in rice, leading to the activation of cold-regulated genes (Ma et al., 2015; Shi et al., 2018). A similar pattern of results was obtained in the present study: a total of nine MAPKs, five CDPKs, six CIPKs, and some RLKs/CRLKs were up-regulated in cultivar NS under freezing stress compared to cultivar NF (Supplementary Table S6). These results suggested that the plasma membrane-located sensors might perceive the signal by the freezing treatment and initiate the Ca^2+^ signaling pathway in rapeseed. Following Ca^2+^ influx to activate the plasma membrane, some protein kinases interact with TFs and trigger downstream freezing-responsive gene expressions.

Phytohormones, including abscisic acid (ABA), jasmonic acid (JA), ethylene (ETH), gibberellin, auxin (IAA), cytokinin, salicylic acid (SA), and brassinosteroid (BR), combine endogenous substances with environmental signals to regulate plant growth and development as well as defense (Huang et al., 2017a). In many instances, plants respond to environmental stresses by producing amounts of ABA, BR, ETH, JA, and SA (Huang et al., 2016; Hu et al., 2017; Wu et al., 2019). Shibasaki et al. (2009) found that cold stress inhibited the expression and transport of auxin-responsive related genes in root. Similar results were obtained in this study; a total of 87 unique DEGs in NST1_NFT1 were enriched in plant hormone signal transduction pathway (Figure 5A; Supplementary Table S6). Among them, 10 DEGs were up-regulated and involved in ABA signaling, encoding abscisic acid-insensitive proteins, serine/threonine-protein kinase SnRK2 (SnRK), and protein phosphatase 2C (PP2C), whereas three DEGs encoding abscisic acid receptors were down-regulated; four DEGs were up-regulated and engaged in ETH signaling, encoding ethylene-responsive transcription factor2 and ethylene response sensor2, while six DEGs encoding ethylene-responsive transcription factors 1/15 and ethylene-insensitive protein were down-regulated. Interestingly, we found that two key enzymes of ethylene biosynthesis, 1-aminocyclopropane-1-carboxylate synthases and 1-aminocyclopropane-1-carboxylate oxidases, were up-regulated. Therefore, we speculate that there may be at least two different ABA and ethylene metabolic pathways associated with freezing tolerance, and one of them was enhanced. Furthermore, 19 DEGs were up-regulated and participated in JA signaling, encoding jasmonic acid synthetase and TIFY proteins; 28 DEGs (19 up-regulation and nine down-regulation) were found and contributed to auxin signaling, encoding auxin-responsive proteins, auxin transporter proteins (ATP), and GH3 auxin-responsive promoter. These are consistent with what has been found in previous research, which suggested that high auxin expression levels could improve freezing tolerance (Pu et al., 2019). Moreover, one DEG was up-regulated, encoding protein BZR1, which is a plant-specific positive regulator of BR signaling (Yin et al., 2005; Li et al., 2017). BRs are perceived by brassinosteroid insensitive1 (BRI1), which cooperates with BRI1-associated receptor kinase1 and positively modulates BZR1 (She et al., 2011; Fang et al., 2019). Fortunately, a leucine-rich repeat receptor-like kinase was found in this study, encoded by BRI1 gene, which was up-regulated. Therefore, we speculated that BZR1 and BRI1 work together to actively mediate BR signaling in response to freezing stress. Apart from that, a large number of studies have shown that BRs can enhance photosynthesis and the activities of antioxidant enzymes (Zhou et al., 2014; Xia et al., 2018). A similar conclusion was reached by the determination of photosynthesis and ROS-scavenging enzymes (Figures 1B, 2). Simultaneously, other DEGs (12 up-regulation and three down-regulation) representing 13 transcription regulators, one gibberellin receptor, and one xyloglucan endotransglucosylase/hydrolase, which plays a crucial role in plant cell wall remodeling (Witasari et al., 2019), were also identified in our study. Taken together, these results indicated that phytohormones play critical roles in response to the freezing stress of rapeseed.

Plant lipoxygenases are a kind of fatty acid dioxygenase with diverse functions and catalyze the peroxidation of polyunsaturated fatty acids such as linolenic and linoleic acids. It was reported that biotic and abiotic stresses cause the release of *α*-linolenic acid, which fascinated particular attention because it represents an essential precursor of jasmonic acid biosynthesis (Yan et al., 2013; Zhao et al., 2014). The synthesis of jasmonic acid begins with the peroxidation of α-linolenic acid by lipoxygenase, the product of which is converted to 12,13-epoxyoctadecatrienoic acid (12,13-EOT) by the allene oxide synthase (AOS). Subsequently, the 12,13-EOT is processed into 12-oxophytodienoic acid (OPDA) by the allene oxide cyclase (AOC), and OPDA is reduced by the OPDA reductase. In this study, all specific DEGs in NST1_NFT1 involved in the alpha-linolenic acid metabolism were found to be up-regulated. Among them, there are nine lipoxygenases, four AOC, two AOS, and seven 12-oxophytodienoate reductases (Figure 5A). Another promising finding was that the alpha-linolenic acid metabolism was also significantly enriched by DAPs, and all DAPs were accumulated in abundance (Figure 5B; Supplementary Table S6). Beyond those and combined with JA signaling resulting from DEGs, these results implied that enhanced jasmonic acid synthesis might regulate the expression of downstream freezing-responsive genes under freezing stress.

Peroxisomes can generate some retrograde signals such as ROS and many metabolites that are important for stress responses (Ng et al., 2014). Glutathione is well known for its ROS scavenging function in biotic stress (Cheng et al., 2015). In this study, six unique DAPs in NST1_NFT1 belonging to the peroxisome pathway were significantly enriched after the freezing treatment (Figure 5B; Supplementary Table S6). Under freezing stress, four catalases with accumulated abundance in cultivar NS compared to cultivar NF were consistent with the determination of CAT enzyme activity (Figure 2A); nevertheless, two peroxidases with reduced abundance in cultivar NS compared to cultivar NF were contrary to the determination of POD enzyme activity (Figure 2C; Supplementary Table S6). This divergence might be attributed to posttranslational modifications (Park et al., 2018). A further novel finding is that the glutathione metabolism pathway was significantly enriched by DAPs, and most of the glutathione S-transferases (GST) enriched in this pathway were accumulated in abundance. In addition, one glutathione peroxidase (GPX) gene was also found to be up-regulated. Our results demonstrated that ROS partook in response to rapeseed freezing tolerance.

Plant secondary metabolites often have no fundamental role in the maintenance of plant life processes, but they are important for the plant to interrelate to its environment for adaptation and defense (Ramakrishna and Ravishankar, 2011). Phenylpropanoid compounds congregate a large family of secondary metabolites that originate from phenylalanine and are beneficial to defense against various stresses in plants (Liu et al., 2015). In the present study, there were five unique abundant accumulated DAPs in NST1_NFT1 encoded by caffeic acid O-methyltransferase, shikimate O-hydroxycinnamoyltransferase, and 3-dehydroquinate dehydratase/shikimate dehydrogenase and implicated in phenylpropanoid biosynthesis (Figure 5B; Supplementary Table S6). It is in agreement with those reported by Zeng et al. (2018) and Pu et al. (2019). In addition, we also found some unique up-regulated DEGs encoding S-adenosylmethionine-dependent methyltransferase, which are involved in the secondary metabolism of many plant species (Ranjan et al., 2020). These results suggested that the accumulated secondary metabolites contribute to the freezing-stressed rapeseed cultivar NS under freezing stress compared to the cultivar NF.

It has been reported that heat shock proteins (HSPs), regarded as the target genes of HSFs, are correlated with cold stress tolerance in grasses (Wang et al., 2016). Similarly, Wang et al. indicated that a large amount of HSPs and chaperone proteins (CPs) was induced by cold stress (Wang et al., 2004). Scarpeci et al. (2008) suggested that ROS, through a direct interaction with HSFs, mediate cellular signals during abiotic stress. In the present study, some up-regulated HSFs, HSPs, and CPs are accompanied with high levels of antioxidation enzyme activities (Figure 2), thus implying that the HSFs might interact with ROS signals to regulate the expression of HSPs and CPs during cold stress. Except the HSPs and CPs, some other unique DEGs encoding cell division control protein, endoplasmin, and protein disulfide isomerase involved in protein synthesis were discovered in NST1_NFT1, and they were up-regulated. In addition, we found some unique up-regulated DEGs in NST1_NFT1, which encode some mitochondria-localized ATP synthase subunit (ASS), cytochrome c oxidase (CCO), and persulfide dioxygenase (PDO). It is known that ASS is the structural basis of proton transportation and energy generation in the mitochondria (Klusch et al., 2017). CCO is the last respiratory complex of the electron transport chain in mitochondria and is responsible for transferring electrons to the final acceptor oxygen in respiratory metabolism (Dahan et al., 2014). The up-regulated ASS and CCO insinuated the increase of energy requirement during leaf development under freezing stress.

### A Possible Freezing Stress-Responsive Molecular Network in Winter Rapeseed

**Figure 8:**
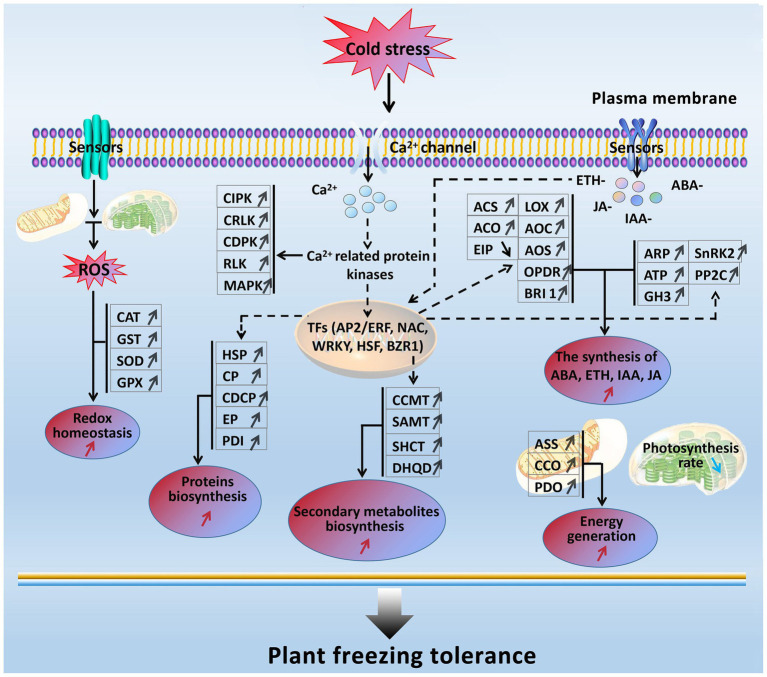
A presumed model for freezing tolerance mechanism in developing rapeseed leaves under freezing stress. The freezing-responsive genes and proteins up-regulated are marked by “↑,” and those down-regulated are marked by “↓.” The enhanced metabolic pathways and cellular processes are marked with the red “↑,” while the reduced ones are marked with the blue “↓.” Dashed arrows indicate possible regulation.

In the present study, based on the transcriptomics and proteomics, a freezing stress-responsive molecular network was generated from the most of significantly enriched freezing-responsive genes and proteins in developing rapeseed leaves (Figure 8). This network covers five foremost functional components, including balancing between ROS production and scavenging, increased signal transduction and transcriptional regulation, enhanced phytohormone (ETH, ABA, IAA, JA, and BR) biosynthesis, accelerated biosynthesis of proteins and secondary metabolites, abundant energy supply, and impaired photosynthesis. Such a molecular network allows us to further understand the principle of freezing tolerance in freezing-treated rapeseed leaves, and some of them can be used as selection index in freezing tolerance breeding program in rapeseed.

When rapeseed plants are exposed to freezing stress, some plasma membrane-located sensors mobilize the signals such as Ca^2+^, ROS, and plant hormones by signal transduction pathways; following these signal influxes, their related protein kinases are activated to interact with TFs and trigger the expression of downstream freezing-tolerant genes. Under persistent freezing stress, the balance between ROS production and scavenging in the cells of rapeseed plants is disrupted. To eliminate the oxidative damage resulting from increased ROS production in mesophyll cells, the rapeseed plant leaves would strive to maintain cellular redox homeostasis by elevating the biosynthesis of antioxidation enzymes (GST, CAT, SOD, and GPX; Figure 2); under freezing stress, the rapeseed plant leaves increase the expression of key enzymes (ASS, CCO, and PDO) in the energy metabolism pathway in response to freezing stress; under freezing stress, the rapeseed plant leaves also heighten the Ca^2+^ signal pathway by up-regulation of MAPK, CDPK, SnRK2, CIPK, RLK, and CRLK. Subsequently, these up-regulated protein kinase genes incorporate with TFs to cause changes in metabolism pathways (enhanced synthesis of proteins and secondary metabolites and increased plant hormone synthesis), suggesting that the production of plant hormones and secondary metabolites may be positively contributing to freezing tolerance. However, these findings need to be demonstrated by further studies.

Furthermore, freezing-tolerant cultivar NS possesses higher ROS scavenging capacity, more stable cell morphology, more ETH, JA, IAA, and ABA production, and stronger photosynthesis and energy supply than freezing-sensitive cultivar NF under freezing stress, which may be the major reasons why cultivar NS is more freezing tolerant than cultivar NF.

## Conclusion

Under freezing stress, the rapeseed cultivar more tolerant to freezing stress would produce stronger signals (Ca^2+^-mediated, ROS, ABA, ETH, JA, IAA, and BR), metabolic pathways (photosynthesis, protein biosynthesis, and secondary metabolite synthesis), and ROS scavenging capacity, more stable cell morphology, and a lower level of lipid peroxidation in leaves.

## Data Availability Statement

The original contributions presented in the study are publicly available. This data can be found here: The sequenced transcriptome raw data have been deposited to the SRA at NCBI with the accession number of PRJNA685002. The mass spectrometry data have been submitted into a public iProX database (Ma et al., 2019) with the accession number of IPX0002682000.

## Author Contributions

The work presented here was carried out in collaboration among all authors. JW executed the omics data analysis and wrote the manuscript. GZ cultivated all plant samples. XY polished and revised this paper. SL performed the analysis of physiological and biochemical characteristics. XD and XC participated in the determination of physiological and biochemical characteristics. HL and XF were engaged in the measurement of photosynthesis. JJ and WM carried out the protein data analysis. ZL designed the experiments. All authors contributed to the article and approved the submitted version.

### Conflict of Interest

The authors declare that the research was conducted in the absence of any commercial or financial relationships that could be construed as a potential conflict of interest.

## Footnotes

^1^http://www.r-project.org/

^2^http://geneontology.org/

^3^http://www.kegg.jp/

## Supplementary Material

Supplementary Figure S1 The reproducibility analysis between three biological replicates of NFT0, NFT1, NST0, and NST1 samples in transcriptome **(A)** and proteome **(B)**, respectively.

Supplementary Figure S2 Pearson’s correlation coefficient between three biological replicates of NFT0, NFT1, NST0, and NST1 samples in transcriptome.

Supplementary Figure S3 Pearson’s correlation coefficient between three biological replicates of NFT0, NFT1, NST0, and NST1 samples in proteome.

Supplementary Figure S4 Gene Ontology classification analysis of the unique differentially expressed genes **(A)** and differentially abundant proteins **(B)** identified in NST1_NFT1.

## References

[ref1] Baxter A. Mittler R. Suzuki N. (2014). ROS as key players in plant stress signalling. J. Exp. Bot. 65, 1229–1240. 10.1093/jxb/ert375, PMID: 24253197

[ref2] Binns D. Dimmer E. Huntley R. Barrell D. O’Donovan C. Apweiler R. (2009). QuickGO: a web-based tool for Gene Ontology searching. Bioinformatics 25, 3045–3046. 10.1093/bioinformatics/btp536, PMID: 19744993 PMC2773257

[ref3] Bruderer R. Bernhardt O. M. Gandhi T. Miladinović S. M. Cheng L. Y. Messner S.. (2015). Extending the limits of quantitative proteome profiling with data-independent acquisition and application to acetaminophen-treated three-dimensional liver microtissues. Mol. Cell. Proteomics 14, 1400–1410. 10.1074/mcp.M114.044305, PMID: 25724911 PMC4424408

[ref4] Chen M. X. Zhu F. Y. Wang F. Z. Ye N. H. Gao B. Chen X.. (2019). Alternative splicing and translation play important roles in hypoxic germination in rice. J. Exp. Bot. 70, 817–833. 10.1093/jxb/ery393, PMID: 30535157 PMC6363088

[ref5] Cheng M. C. Ko K. Chang W. L. Kuo W. C. Chen G. H. Lin T. P. (2015). Increased glutathione contributes to stress tolerance and global translational changes in *Arabidopsis*. Plant J. 83, 926–939. 10.1111/tpj.12940, PMID: 26213235

[ref6] Chinnusamy V. Zhu J. Zhu J. K. (2007). Cold stress regulation of gene expression in plants. Trends Plant Sci. 12, 444–451. 10.1016/j.tplants.2007.07.002, PMID: 17855156

[ref7] Chu P. Yan G. X. Yang Q. Zhai L. N. Zhang C. Zhang F. Q.. (2015). iTRAQ-based quantitative proteomics analysis of *Brassica napus* leaves reveals pathways associated with chlorophyll deficiency. J. Proteome 113, 244–259. 10.1016/j.jprot.2014.10.005, PMID: 25317966

[ref8] Dahan J. Tcherkez G. Macherel D. Benamar A. Belcram K. (2014). Disruption of the CYTOCHROME C OXIDASE DEFICIENT1 gene leads to cytochrome c oxidase depletion and reorchestrated respiratory metabolism in *Arabidopsis*. Plant Physiol. 166, 1788–1802. 10.1104/pp.114.248526, PMID: 25301889 PMC4256860

[ref9] Deng S. Sun J. Zhao R. Ding M. Zhang Y. Sun Y.. (2015). Populus euphratica APYRASE2 enhances cold tolerance by modulating vesicular trafficking and extracellular ATP in *Arabidopsis* plants. Plant Physiol. 169, 530–548. 10.1104/pp.15.00581, PMID: 26224801 PMC4577398

[ref10] Ding Y. Li H. Zhang X. Xie Q. Gong Z. Yang S. (2015). OST1 kinase modulates freezing tolerance by enhancing ICE1 stability in *Arabidopsis*. Dev. Cell 32, 278–289. 10.1016/j.devcel.2014.12.023, PMID: 25669882

[ref11] Ding Y. Shi Y. Yang S. (2019). Advances and challenges in uncovering cold tolerance regulatory mechanisms in plants. New Phytol. 222, 1690–1704. 10.1111/nph.15696, PMID: 30664232

[ref12] Egertson J. D. Kuehn A. Merrihew G. E. Bateman N. W. MacLean B. X. Ting Y. S.. (2013). Multiplexed MS/MS for improved data-independent acquisition. Nat. Methods 10, 744–756. 10.1038/nmeth.2528, PMID: 23793237 PMC3881977

[ref13] Fang P. Yan M. Chi C. Wang M. Zhou Y. Zhou J.. (2019). Brassinosteroids act as a positive regulator of photoprotection in response to chilling stress. Plant Physiol. 180, 2061–2076. 10.1104/pp.19.00088, PMID: 31189657 PMC6670110

[ref14] Gao F. Ma P. Wu Y. Zhou Y. Zhang G. (2019). Quantitative proteomic analysis of the response to cold stress in *Jojoba*, a tropical woody crop. Int. J. Mol. Sci. 20:243. 10.3390/ijms20020243, PMID: 30634475 PMC6359463

[ref15] Hu Y. Jiang Y. Han X. Wang H. Pan J. Yu D. (2017). Jasmonate regulates leaf senescence and tolerance to cold stress: crosstalk with other phytohormones. J. Exp. Bot. 68, 1361–1369. 10.1093/jxb/erx004, PMID: 28201612

[ref16] Huang H. Liu B. Liu L. Song S. (2017a). Jasmonate action in plant growth and development. J. Exp. Bot. 68, 1349–1359. 10.1093/jxb/erw495, PMID: 28158849

[ref17] Huang Y. C. Niu C. Y. Yang C. R. Jinn T. L. (2016). The heat stress factor HSFA6b connects ABA signaling and ABA-mediated heat responses. Plant Physiol. 172, 1182–1199. 10.1104/pp.16.00860, PMID: 27493213 PMC5047099

[ref18] Huang X. B. Shi H. Y. Hu Z. R. Liu A. Amombo E. Chen L.. (2017b). ABA is involved in regulation of cold stress response in bermudagrass. Front. Plant Sci. 8:1613. 10.3389/fpls.2017.01613, PMID: 29081782 PMC5645512

[ref19] Jia Y. Ding Y. Shi Y. Zhang X. Gong Z. Yang S. (2016). The *cbf*s triple mutants reveal the essential functions of CBFs in cold acclimation and allow the definition of CBF regulons in *Arabidopsis*. New Phytol. 212, 345–353. 10.1111/nph.14088, PMID: 27353960

[ref20] Jiang B. Shi Y. Zhang X. Xin X. Qi L. Guo H.. (2017). PIF3 is a negative regulator of the CBF pathway and freezing tolerance in *Arabidopsis*. Proc. Natl. Acad. Sci. U. S. A. 114, 6695–6702. 10.1073/pnas.1706226114, PMID: 28739888 PMC5559041

[ref21] Kanehisa M. Sato Y. Kawashima M. Furumichi M. Tanabe M. (2016). KEGG as a reference resource for gene and protein annotation. Nucleic Acids Res. 44, 457–462. 10.1093/nar/gkv1070, PMID: 26476454 PMC4702792

[ref22] Kaplan F. Kopka J. Sung D. Y. Zhao W. Popp M. Porat R.. (2007). Transcript and metabolite profiling during cold acclimation of *Arabidopsis* reveals an intricate relationship of cold-regulated gene expression with modifications in metabolite content. Plant J. 50, 967–981. 10.1111/j.1365-313X.2007.03100.x, PMID: 17461790

[ref23] Kim S. H. Kim H. S. Bahk S. An J. Yoo Y. Kim J. Y.. (2017). Phosphorylation of the transcriptional repressor MYB15 by mitogen-activated protein kinase 6 is required for freezing tolerance in *Arabidopsis*. Nucleic Acids Res. 45, 6613–6627. 10.1093/nar/gkx417, PMID: 28510716 PMC5499865

[ref24] Kim Y. Park S. Gilmour S. J. Thomashow M. F. (2013a). Roles of CAMTA transcription factors and salicylic acid in configuring the low-temperature transcriptome and freezing tolerance of *Arabidopsis*. Plant J. 75, 364–376. 10.1111/tpj.12205, PMID: 23581962

[ref25] Kim D. Pertea G. Trapnell C. Pimentel H. Kelley R. Salzberg S. L. (2013b). TopHat2: accurate alignment of transcriptomes in the presence of insertions, deletions and gene fusions. Genome Biol. 14:36. 10.1186/gb-2013-14-4-r36, PMID: 23618408 PMC4053844

[ref26] Klusch N. Murphy B. J. Mills D. J. Yildiz O. Kuhlbrandt W. (2017). Structural basis of proton translocation and force generation in mitochondrial ATP synthase. Elife 6:e33274. 10.7554/eLife.33274, PMID: 29210357 PMC5747523

[ref27] Li H. Ye K. Shi Y. Cheng J. Zhang X. Yang S. (2017). BZR1 positively regulates freezing tolerance via CBF-dependent and CBF-independent pathways in *Arabidopsis*. Mol. Plant 10, 545–559. 10.1016/j.molp.2017.01.004, PMID: 28089951

[ref28] Liu Z. Jia Y. Ding Y. Shi Y. Li Z. Guo Y.. (2017). Plasma membrane CRPK1-mediated phosphorylation of 14-3-3 proteins induces their nuclear import to fine-tune CBF signaling during cold response. Mol. Cell 66, 117–128. 10.1016/j.molcel.2017.02.016, PMID: 28344081

[ref29] Liu J. Osbourn A. Ma P. (2015). MYB transcription factors as regulators of phenylpropanoid metabolism in plants. Mol. Plant 8, 689–708. 10.1016/j.molp.2015.03.012, PMID: 25840349

[ref30] Livanos P. Galatis B. Apostolakos P. (2014). The interplay between ROS and tubulin cytoskeleton in plants. Plant Signal. Behav. 9:e28069. 10.4161/psb.28069, PMID: 24521945 PMC4091245

[ref31] Ma J. Chen T. Wu S. Yang C. Bai M. Shu K.. (2019). iProX: an integrated proteome resource. Nucleic Acids Res. 47, 1211–1217. 10.1093/nar/gky869, PMID: 30252093 PMC6323926

[ref32] Ma Y. Dai X. Xu Y. Luo W. Zheng X. Zeng D.. (2015). COLD1 confers chilling tolerance in rice. Cell 160, 1209–1221. 10.1016/j.cell.2015.01.046, PMID: 25728666

[ref33] Mi W. Liu Z. Jin J. Dong X. Xu C. Zou Y.. (2021). Comparative proteomics analysis reveals the molecular mechanism of enhanced cold tolerance through ROS scavenging in winter rapeseed (*Brassica napus* L.). PLoS One 16:e0243292. 10.1371/journal.pone.0243292, PMID: 33434207 PMC7802968

[ref34] Ng S. De Clercq I. Van Aken O. Law S. R. Ivanova A. Willems P.. (2014). Anterograde and retrograde regulation of nuclear genes encoding mitochondrial proteins during growth, development, and stress. Mol. Plant 7, 1075–1093. 10.1093/mp/ssu037, PMID: 24711293

[ref35] Park S. Lee C. M. Doherty C. J. Gilmour S. J. Kim Y. Thomashow M. F. (2015). Regulation of the *Arabidopsis* CBF regulon by a complex low temperature regulatory network. Plant J. 82, 193–207. 10.1111/tpj.12796, PMID: 25736223

[ref36] Park J. Lim C. J. Shen M. Park H. J. Cha J. Y. Iniesto E.. (2018). Epigenetic switch from repressive to permissive chromatin in response to cold stress. Proc. Natl. Acad. Sci. U. S. A. 115, 5400–5409. 10.1073/pnas.1721241115, PMID: 29784800 PMC6003311

[ref37] Pu Y. Liu L. Wu J. Zhao Y. Bai J. Ma L.. (2019). Transcriptome profile analysis of winter rapeseed (*Brassica napus* L.) in response to freezing stress, reveal potentially connected events to freezing stress. Int. J. Mol. Sci. 20:2771. 10.3390/ijms20112771, PMID: 31195741 PMC6600501

[ref38] Ramakrishna A. Ravishankar G. A. (2011). Influence of abiotic stress signals on secondary metabolites in plants. Plant Signal. Behav. 6, 1720–1731. 10.4161/psb.6.11.17613, PMID: 22041989 PMC3329344

[ref39] Ranjan R. Kumar N. Gautam A. Dubey A. K. Pandey S. N. Mallick S. (2020). Chlorella sp. modulates the glutathione mediated detoxification and S-adenosylmethionine dependent methyltransferase to counter arsenic toxicity in *Oryza sativa* L. Ecotoxicol. Environ. Saf. 208:111418. 10.1016/j.ecoenv.2020.111418, PMID: 33045435

[ref40] Ray P. D. Huang B. W. Tsuji Y. (2012). Reactive oxygen species (ROS) homeostasis and redox regulation in cellular signaling. Cell. Signal. 24, 981–990. 10.1016/j.cellsig.2012.01.008, PMID: 22286106 PMC3454471

[ref41] Scarpeci T. E. Zanor M. I. Valle E. M. (2008). Investigating the role of plant heat shock proteins during oxidative stress. Plant Signal. Behav. 3, 856–857. 10.4161/psb.3.10.6021, PMID: 19704521 PMC2634396

[ref42] She J. Han Z. Kim T. W. Wang J. Cheng W. Chang J.. (2011). Structural insight into brassinosteroid perception by BRI1. Nature 474, 472–476. 10.1038/nature10178, PMID: 21666666 PMC4019668

[ref43] Shi Y. Ding Y. Yang S. (2018). Molecular regulation of CBF signaling in cold acclimation. Trends Plant Sci. 23, 623–637. 10.1016/j.tplants.2018.04.002, PMID: 29735429

[ref44] Shi Y. Tian S. Hou L. Huang X. Zhang X. Guo H.. (2012). Ethylene signaling negatively regulates freezing tolerance by repressing expression of CBF and type-A ARR genes in *Arabidopsis*. Plant Cell 24, 2578–2595. 10.1105/tpc.112.098640, PMID: 22706288 PMC3406918

[ref45] Shibasaki K. Uemura M. Tsurumi S. Rahman A. (2009). Auxin response in *Arabidopsis* under cold stress: underlying molecular mechanisms. Plant Cell 21, 3823–3838. 10.1105/tpc.109.069906, PMID: 20040541 PMC2814496

[ref46] Toyooka K. Sato M. Kutsuna N. Nagata N. (2014). Development of high resolution TEM image acquisition system by using high-pressure freezing method. Plant Morphol. 26, 3–8. 10.5685/plmorphol.26.3

[ref47] Trapnell C. Williams B. A. Pertea G. Mortazavi A. Kwan G. van Baren M. J.. (2010). Transcript assembly and quantification by RNA-Seq reveals unannotated transcripts and isoform switching during cell differentiation. Nat. Biotechnol. 28, 511–515. 10.1038/nbt.1621, PMID: 20436464 PMC3146043

[ref48] Wang Y. Dai Y. Tao X. Wang J. Z. Cheng H. Y. Yang H.. (2016). Heat shock factor genes of tall fescue and perennial ryegrass in response to temperature stress by RNA-Seq analysis. Front. Plant Sci. 6:1226. 10.3389/fpls.2015.01226, PMID: 26793208 PMC4707269

[ref49] Wang W. Vinocur B. Shoseyov O. Altman A. (2004). Role of plant heat-shock proteins and molecular chaperones in the abiotic stress response. Trends Plant Sci. 9, 244–252. 10.1016/j.tplants.2004.03.006, PMID: 15130550

[ref50] Wei J. Liu X. Li L. Zhao H. Liu S. Yu X.. (2020). Quantitative proteomic, physiological and biochemical analysis of cotyledon, embryo, leaf and pod reveals the effects of high temperature and humidity stress on seed vigor formation in soybean. BMC Plant Biol. 20:127. 10.1186/s12870-020-02335-1, PMID: 32216758 PMC7098090

[ref51] Witasari L. D. Huang F. C. Hoffmann T. Rozhon W. Fry S. C. Schwab W. (2019). Higher expression of the strawberry xyloglucan endotransglucosylase/hydrolase genes FvXTH9 and FvXTH6 accelerates fruit ripening. Plant J. 100, 1237–1253. 10.1111/tpj.14512, PMID: 31454115 PMC8653885

[ref52] Wu Z. Han S. Zhou H. Tuang Z. K. Wang Y. Jin Y.. (2019). Cold stress activates disease tolerance in *Arabidopsis thaliana* through a salicylic acid dependent pathway. Plant Cell Environ. 42, 2645–2663. 10.1111/pce.13579, PMID: 31087367

[ref53] Xia X. J. Fang P. P. Guo X. Qian X. J. Zhou J. Shi K.. (2018). Brassinosteroid-mediated apoplastic H_2_O_2_-glutaredoxin 12/14 cascade regulates antioxidant capacity in response to chilling in tomato. Plant Cell Environ. 41, 1052–1064. 10.1111/pce.13052, PMID: 28776692

[ref54] Xie Y. Chen P. Yan Y. Bao C. Li X. Wang L.. (2018). An atypical R2R3 MYB transcription factor increases cold hardiness by CBF-dependent and CBF-independent pathways in apple. New Phytol. 218, 201–218. 10.1111/nph.14952, PMID: 29266327

[ref55] Xu Y. Zeng X. Wu J. Zhang F. Li C. Jiang J.. (2018). iTRAQ-based quantitative proteome revealed metabolic changes in winter turnip rape (*Brassica rapa* L.) under cold stress. Int. J. Mol. Sci. 19:3346. 10.3390/ijms19113346, PMID: 30373160 PMC6274765

[ref56] Yan L. Zhai Q. Wei J. Li S. Wang B. Huang T.. (2013). Role of tomato lipoxygenase D in wound-induced jasmonate biosynthesis and plant immunity to insect herbivores. PLoS Genet. 9:e1003964. 10.1371/journal.pgen.1003964, PMID: 24348260 PMC3861047

[ref57] Yin Y. Vafeados D. Tao Y. Yoshida S. Asami T. Chory J. (2005). A new class of transcription factors mediates brassinosteroid-regulated gene expression in *Arabidopsis*. Cell 120, 249–259. 10.1016/j.cell.2004.11.044, PMID: 15680330

[ref58] Zeng X. Xu Y. Jiang J. Zhang F. Ma L. Wu D.. (2018). iTRAQ-based comparative proteomic analysis of the roots of two winter turnip rapes (*Brassica rapa* L.) with different freezing-tolerance. Int. J. Mol. Sci. 19:4077. 10.3390/ijms19124077, PMID: 30562938 PMC6321220

[ref59] Zhang Z. Liu H. Sun C. Ma Q. Bu H. Chong K.. (2018). A C2H2 zinc-finger protein OsZFP213 interacts with OsMAPK3 to enhance salt tolerance in rice. J. Plant Physiol. 229, 100–110. 10.1016/j.jplph.2018.07.003, PMID: 30055519

[ref60] Zhang C. Y. Zhang Z. Y. Li J. H. Li F. Liu H. H. Yang W. S.. (2017). OsMAPK3 phosphorylates OsbHLH002/OsICE1 and inhibits its ubiquitination to activate OsTPP1 and enhances rice chilling tolerance. Dev. Cell 43, 731–743. 10.1016/j.devcel.2017.11.016, PMID: 29257952

[ref61] Zhao Y. Dong W. Zhang N. Ai X. Wang M. Huang Z.. (2014). A wheat allene oxide cyclase gene enhances salinity tolerance via jasmonate signaling. Plant Physiol. 164, 1068–1076. 10.1104/pp.113.227595, PMID: 24326670 PMC3912080

[ref62] Zhao C. Wang P. Si T. Hsu C. C. Wang L. Zayed O.. (2017). MAP kinase cascades regulate the cold response by modulating ICE1 protein stability. Dev. Cell 43, 618–629. 10.1016/j.devcel.2017.09.024, PMID: 29056551 PMC5716877

[ref63] Zhao C. Zhang Z. Xie S. Si T. Li Y. Zhu J. K. (2016). Mutational evidence for the critical role of CBF transcription factors in cold acclimation in *Arabidopsis*. Plant Physiol. 171, 2744–2759. 10.1104/pp.16.00533, PMID: 27252305 PMC4972280

[ref64] Zhou J. Wang J. Li X. Xia X. J. Zhou Y. H. Shi K.. (2014). H_2_O_2_ mediates the crosstalk of brassinosteroid and abscisic acid in tomato responses to heat and oxidative stresses. J. Exp. Bot. 65, 4371–4383. 10.1093/jxb/eru217, PMID: 24899077 PMC4112640

[ref65] Zhu J. (2016). Review abiotic stress signaling and responses in plants. Cell 167, 313–324. 10.1016/j.cell.2016.08.029, PMID: 27716505 PMC5104190

