# Supplementary figures and images for: Comparative Transcriptomics and Proteomics Analyses of Leaves Reveals a Freezing Stress-Responsive Molecular Network in Winter Rapeseed (Brassica rapa L.)
Source: Front Plant Sci. 2021 Apr 28;12:664311. doi: 10.3389/fpls.2021.664311 (PMC8113625; doi:10.3389/fpls.2021.664311)

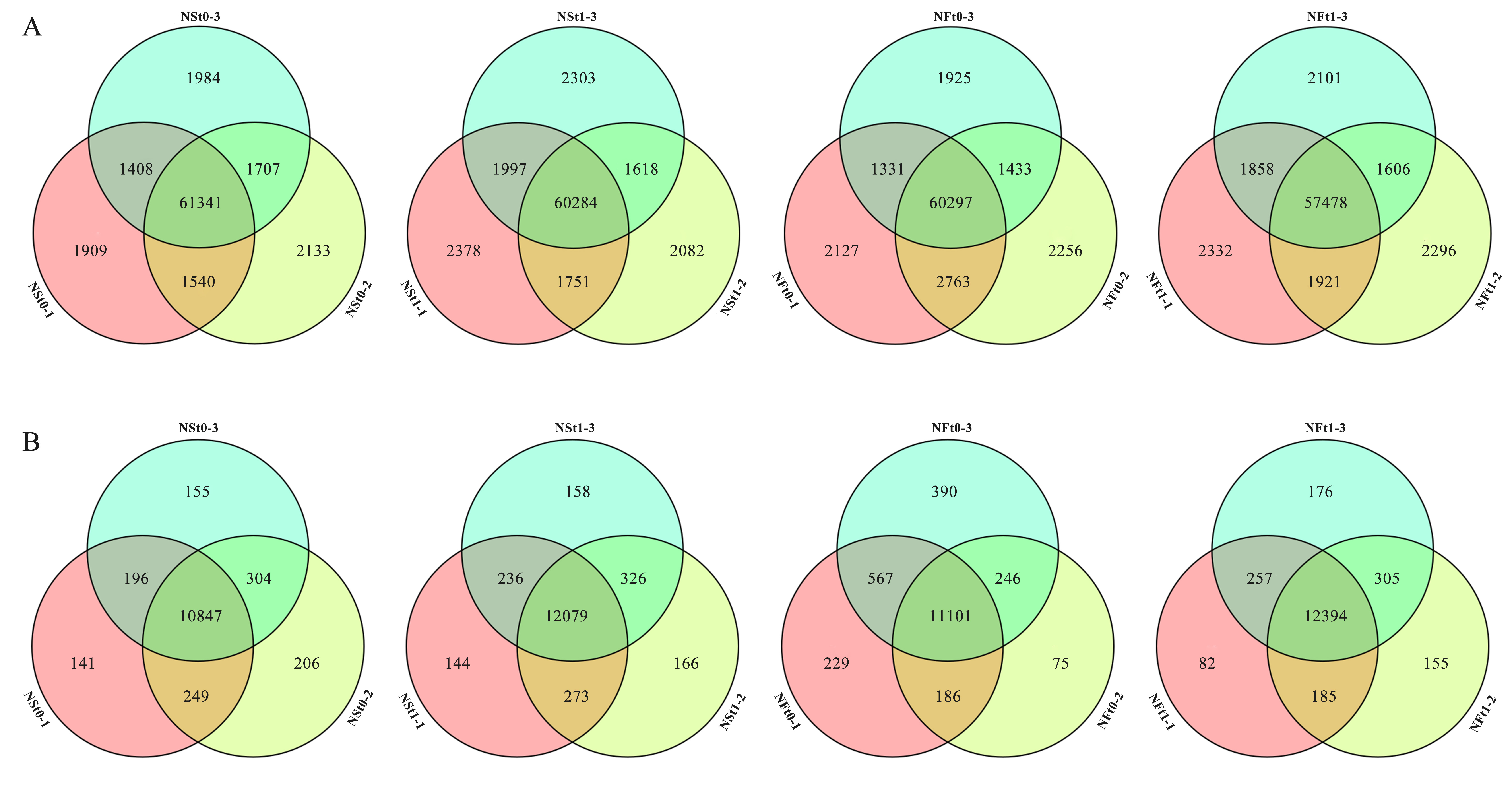

Supplement: Supplementary Figure S1 — The reproducibility analysis between three biological replicates of NFT0, NFT1, NST0, and NST1 samples in transcriptome (A) and proteome (B), respectively. [file Data_Sheet_1.ZIP › Additional files/Fig S1.jpg]

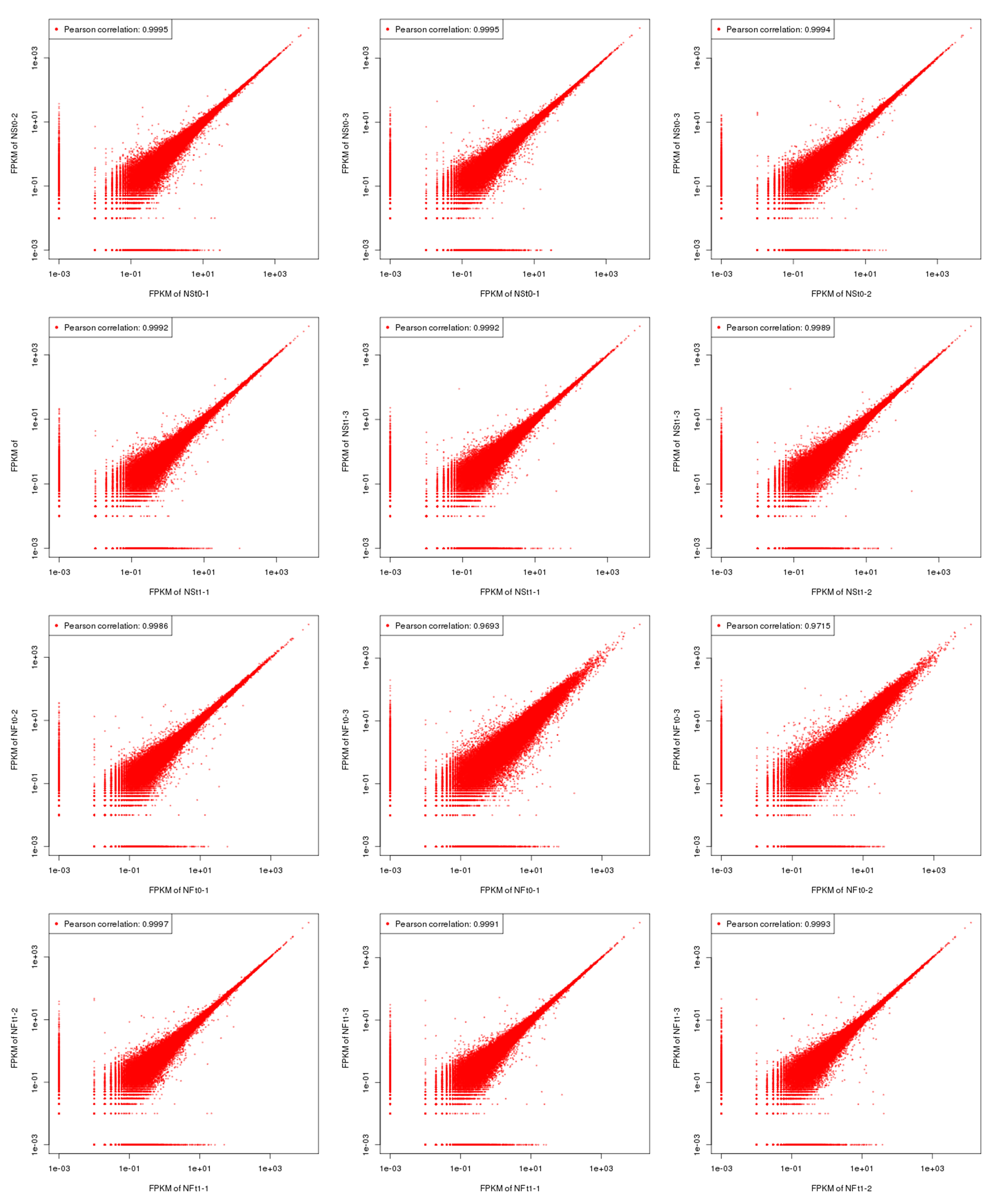

Supplement: Supplementary Figure S1 — The reproducibility analysis between three biological replicates of NFT0, NFT1, NST0, and NST1 samples in transcriptome (A) and proteome (B), respectively. [file Data_Sheet_1.ZIP › Additional files/Fig S2.jpg]

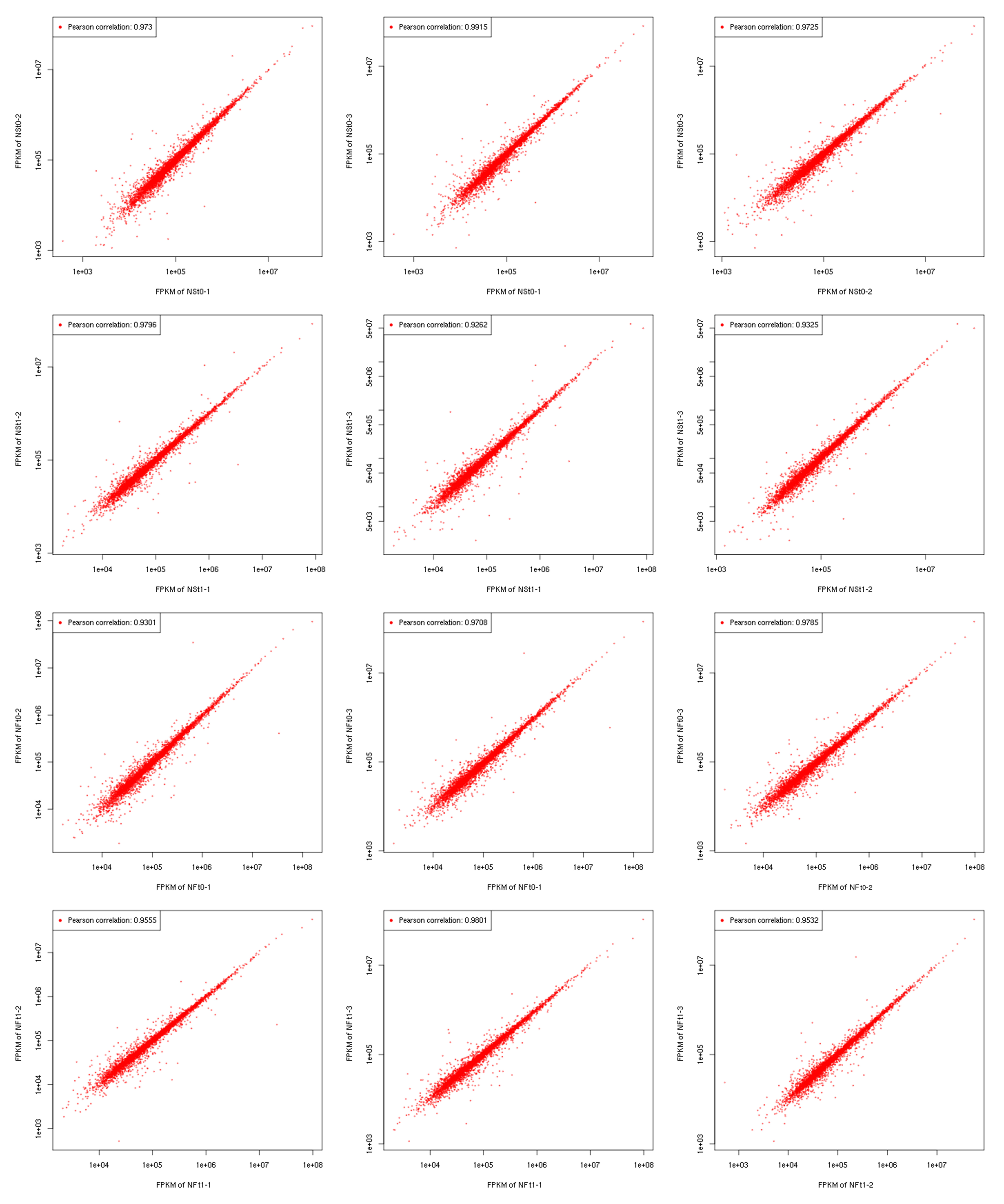

Supplement: Supplementary Figure S1 — The reproducibility analysis between three biological replicates of NFT0, NFT1, NST0, and NST1 samples in transcriptome (A) and proteome (B), respectively. [file Data_Sheet_1.ZIP › Additional files/Fig S3.jpg]

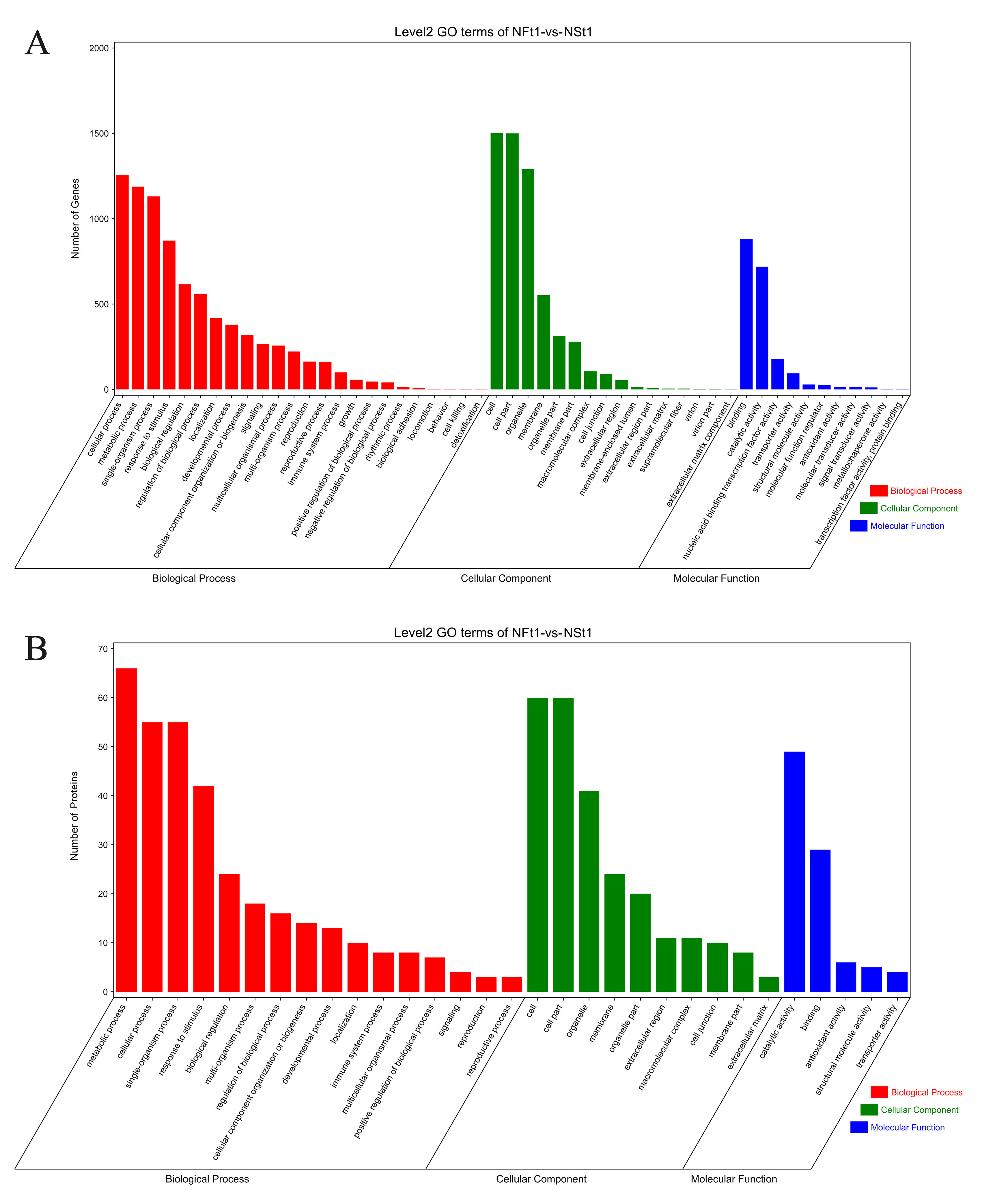

Supplement: Supplementary Figure S1 — The reproducibility analysis between three biological replicates of NFT0, NFT1, NST0, and NST1 samples in transcriptome (A) and proteome (B), respectively. [file Data_Sheet_1.ZIP › Additional files/Fig S4.jpg]
